# Role of ICAM-1 in impaired retinal circulation in rhegmatogenous retinal detachment

**DOI:** 10.1038/s41598-021-94993-w

**Published:** 2021-07-28

**Authors:** Harumasa Yokota, Taiji Nagaoka, Hidetaka Noma, Akemi Ofusa, Tomoe Kanemaki, Hiroshi Aso, Hirotsugu Hanazaki, Satoru Yamagami, Masahiko Shimura

**Affiliations:** 1 Division of Ophthalmology, Department of Visual Sciences, Nihon University School of Medicine, 30-1 Oyaguchi-Kamicho, Itabashi-ku, Tokyo, 173-8610 Japan; 2 Department of Ophthalmology, Hachioji Medical Center, Tokyo Medical University, Tokyo, Japan

**Keywords:** Eye diseases, Retinal diseases

## Abstract

Many studies have demonstrated that rhegmatogenous retinal detachment (RRD) leads to impaired retinal circulation. However, the involvement of inflammation in the RRD-induced worsening of retinal circulation was obscure. This retrospective observational study included 150 patients with primary RRD (macula-on, n = 63; macula-off, n = 87) who underwent 25-gauge microincision vitrectomy surgery (25G MIVS). Total retinal blood flow was represented by the mean blur rate (MBR) of the optic nerve head vessel, measured by laser speckle flowgraphy preoperatively and until 6 months postoperatively. Aqueous humor samples were obtained during surgery to determine cytokine concentrations by enzyme-linked immunosorbent assay. At 3 and 6 months postoperatively, there were no significant differences between eyes with macula-on RRD and fellow eyes. However, in macula-off RRD, MBR remained significantly lower in RRD eyes 6 months postoperatively (*P* < 0.05). Log-transformed levels of soluble intercellular adhesion molecule-1 (sICAM-1) were negatively correlated with relative MBR (r-MBR, RRD eye/fellow eye) before surgery (*r* =  − 0.47, *P* = 0.01) in macula-on, but not macula-off, RRD. Six months postoperatively, r-MBR correlated significantly with sICAM-1 levels (*r* =  − 0.36, *P* = 0.02) in macula-off RRD. ICAM-1 may play a role in RRD-induced deterioration of retinal circulation.

## Introduction

Rhegmatogenous retinal detachment (RRD) is an acute retinal disorder characterized by the mechanical separation of the neurosensory retina from the retinal pigment epithelium. Since photoreceptor cells in the neurosensory retina are supported by retinal pigment epithelium cells, RRD usually induces photoreceptor apoptosis^1^. Therefore, prompt repair is required to prevent photoreceptor cell damage after RRD. In general, surgical repair is by pars plana vitrectomy (PPV) or scleral buckling, both of which obtain high rates of successful reattachment with a single operation^2^. Recently, PPV has become the more popular procedure with the advent of microincision vitrectomy surgery (MIVS), which results in less intraoperative and postoperative pain and a shorter operation time^3^. However, although complete mechanical attachment can be obtained by surgery after RRD, recovery of visual function is often incomplete.

The importance of detachment in the macular area in preoperative RRD is well known. The macula has a high density of photoreceptors, which require substantial nutrition via the retinal and choroidal circulation. Retinal circulation is impaired in eyes with RRD^4–6^ but was reported to recover to the levels in the fellow eyes by about 6 months postoperatively^6,7^. Because conventional methods for measuring ocular circulation are time consuming and partially invasive, it is difficult to repeat measurements to record longitudinal changes in retinal and/or choroidal circulation after surgical repair of RRD.

Recently, laser speckle flowgraphy (LSFG) has been recognized as a useful method of measuring retinal and choroidal circulation non-invasively and without the aid of contrast medium^8–11^. Using LSFG, Iwase et al. examined the longitudinal changes in retinal circulation measured before and after 25-gauge (25G) MIVS or scleral buckling in RRD eyes with the macula still attached (macula-on RRD)^4^. They demonstrated that 25G MIVS, but not scleral buckling, resulted in the recovery of retinal circulation by 6 months postoperatively^4^. However, to our knowledge, no study using LSFG has evaluated longitudinal changes in retinal circulation after surgical repair of RRD that involved the macula (macula-off RRD) in a relatively large cohort.

In RRD eyes, inflammatory cytokines are markedly upregulated and considered a decisive factor in promoting apoptotic death of photoreceptors in the detached retina^12–14^. However, whether intraocular cytokine levels can affect longitudinal changes in retinal circulation after RRD is unknown. Therefore, using LSFG, we compared the longitudinal changes in retinal circulation after macula-on and macula-off RRD treated with 25G MIVS. In addition, we aimed to assess whether the levels of cytokines in the aqueous humor correlated with retinal circulation in postoperative RRD eyes.

## Results

### Participant characteristics

**Figure 1:**
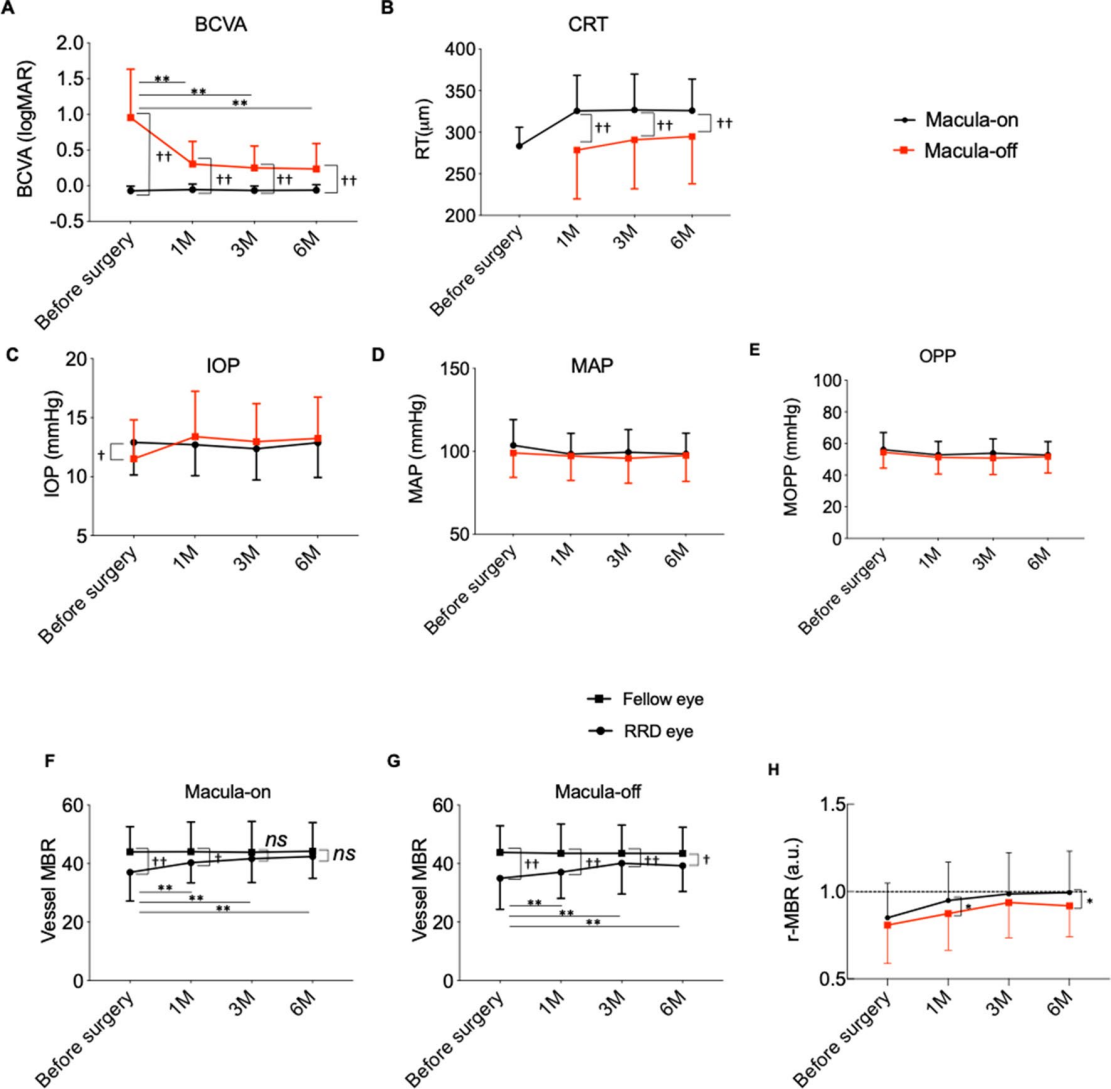
Time course of variables of eyes with rhegmatogenous retinal detachment (RRD). (**A**) Best-corrected visual acuity (BCVA) of RRD eyes was significantly worse in macula-off RRD than in macula-on RRD. (**B**) Central retinal thickness (CRT) was significantly thinner in macula-off RRD than in macula-on RRD. (**C**) Intraocular pressure (IOP) was significantly lower in macula-off RRD than in macula-on RRD before surgery. (**D**) Mean arterial pressure (MAP). (**E**) Ocular perfusion pressure (OPP). (**F**,**G)** Time course of vessel MBR in the optic nerve head in RRD eyes (filled circle) and fellow eyes (filled square, open square) in macula-on RRD (**F**) and macula-off RRD (**G**). (**H**) Comparison of relative MBR (r-MBR, RRD eye/fellow eye) at the optic nerve head. (**A–E**,**H**) **P < 0.01 vs. before surgery; ^†^*P* < 0.05, ^††^*P* < 0.01 between macula-on (black line) and -off (red line) RRD. **P < 0.01 vs. before surgery; ^†^*P* < 0.05, ^††^*P* < 0.01 between RRD and fellow eyes. (**F**,**G**) **P < 0.01 vs. before surgery; ^†^*P* < 0.05, ^††^*P* < 0.01 between RRD and fellow eyes.

Preoperative characteristics of the participants are shown in Table 1. The IOP and diastolic BP were significantly lower in subjects with macula-off RRD than those with macula-on RRD. Time courses of BCVA (logMAR), CRT, IOP, MAP and OPP are shown in Fig. 1A–E, respectively. BCVA was significantly lower in macula-off RRD than in macula-on RRD until at least 6 months after surgery. CRT was also significantly thinner in macula-off RRD than in macula-on RRD until at least 6 months postoperatively. IOP was significantly lower in macula-off RRD than in macula-on RRD at baseline, but this difference disappeared by 1 month postoperatively. No significant changes between visits or differences between groups were observed in MAP or OPP throughout the 6-month observation period.

**Table 1:** Clinical characteristics of participants.

	Macula-on	Macula-off	*P* value
Eyes, n	63	87	–
Age, y	55.0 ± 12.6	58.1 ± 13.4	0.15
Male/female, n	38/25	56/31	0.73
Preoperative IOP, mmHg	12.9 ± 2.7	11.5 ± 3.3	< 0.01
Axial length, mm	25.6 ± 1.6	25.4 ± 1.6	0.54
Systolic blood pressure, mmHg	136.6 ± 20.4	132.9 ± 19.0	0.26
Diastolic blood pressure, mmHg	87.0 ± 14.7	81.9 ± 14.4	< 0.05
Mean arterial pressure, mmHg	103.5 ± 15.5	98.9 ± 14.7	0.07
Ocular perfusion pressure, mmHg	56.0 ± 10.9	54.4 ± 9.9	0.34
PPV/PPV + PEA + IOL, n	14/49	33/54	0.0501

Values are mean ± SEM unless stated otherwise.

*IOP* intraocular pressure, *PPV* pars plana vitrectomy, *PEA* phacoemulsification and aspiration, *IOL* intraocular lens.

### Optic nerve head vessel MBR

At baseline, the vessel MBR in the optic nerve head was significantly lower in RRD eyes than in fellow eyes (Fig. 1F,G). In macula-on RRD, vessel MBR in RRD eyes recovered gradually, reaching the same level as in fellow eyes by 3 months postoperatively. In macula-off RRD, the MBR increased significantly from baseline after surgery in RRD eyes. However, it was still significantly lower than that in fellow eyes 6 months postoperatively. To further confirm the persistent worsening of retinal circulation in macula-off RRD eyes, we compared r-MBR in macula-on and -off RRD eyes (Fig. 1H) and found that a significantly lower ratio remained in the macula-off RRD eyes until at least 6 months postoperatively.

To elucidate whether impaired retinal circulation at baseline creates any harmful effects on ocular parameters 6 months postoperatively, we performed Pearson correlation coefficient analysis. BCVA and CRT at 6 months postoperatively were not correlated with r-MBR before surgery (data not shown).

### Aqueous humor levels of cytokines in RRD eyes

**Figure 2:**
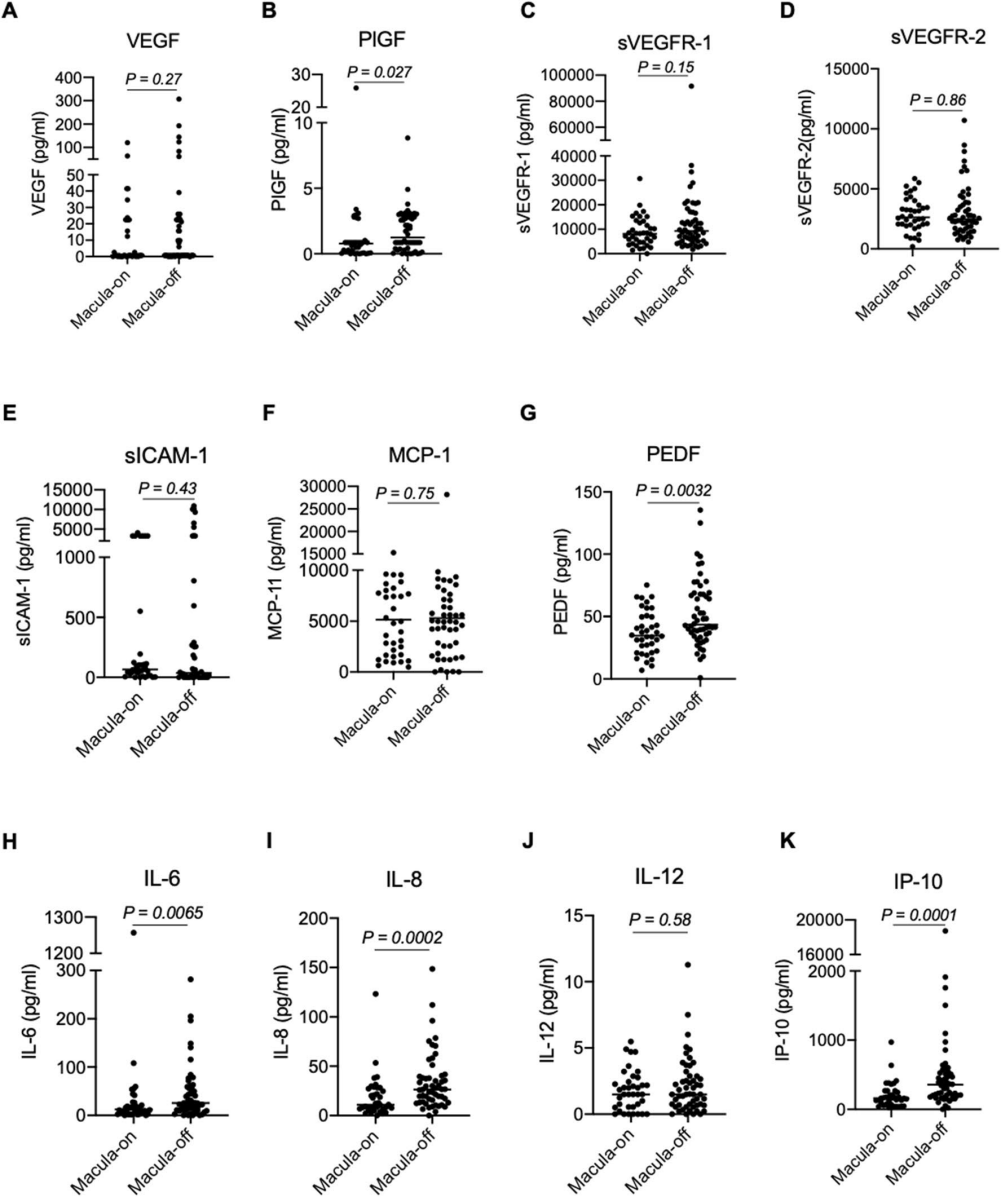
Aqueous humor levels of cytokines in RRD eyes. Vascular endothelial growth factor (VEGF) (**A**), placental growth factor (PlGF) (**B**), soluble vascular endothelial growth factor receptor (sVEGFR)-1 (**C**), sVEGFR-2 (**D**), soluble intercellular adhesion molecule-1 (sICAM-1) (**E**), monocyte chemoattractant protein-1 (MCP-1) (**F**), pigment epithelium-derived factor (PEDF) (**G**), interleukin (IL)-6 (**H**), IL-8 (**I**), IL-12 (**J**), and interferon-inducible 10-kDa protein (IP-10) (**K**) were measured. The levels of PlGF, PEDF, IL-6, IL-8 and IP-10 were significant higher in macula-off RRD than those in macula-on RRD.

We excluded subjects with a blank cytokine measurement, leaving 92 subjects included in this additional analysis (macula-on, 38; macula-off, 54). We compared cytokine concentrations in the aqueous humor of eyes with macula-on and macula-off RRD (Fig. 2). Significantly higher levels of placental growth factor (PlGF), pigment epithelium-derived factor (PEDF), interleukin (IL)-6, IL-8, and interferon-inducible 10-kDa protein (IP-10) were present in the macula-off RRD eyes than in the macula-on RRD eyes. To elucidate whether cytokines influence retinal circulation, we calculated the Pearson correlation coefficient between the log-transformed levels of cytokines at baseline and the r-MBR of RRD eyes to fellow eyes (Table 2). Only soluble intercellular adhesion molecule-1 (sICAM-1) was correlated negatively with r-MBR before surgery (*r* =  − 0.27, *P* = 0.03), and 3 (*r* =  − 0.29, *P* = 0.01) and 6 months after surgery (*r* =  − 0.25, *P* = 0.04).

**Table 2:** Pearson correlation coefficients between cytokine levels in aqueous humor during surgery and r-MBR before surgery and 1, 3, and 6 months after surgery.

Cytokines	Before surgery		After surgery					
			1 month		3 months		6 months	
	*r*	*P* value	*r*	*P* Value	*r*	*P* value	*r*	*P* value
sVEGFR-1	− 0.04	0.63	− 0.09	0.41	− 0.05	0.63	− 0.12	0.26
sVEGFR-2	0.02	0.87	− 0.19	0.22	− 0.05	0.63	− 0.02	0.81
VEGF	− 0.17	0.23	− 0.07	0.50	0.02	0.81	− 0.01	0.91
PlGF	− 0.02	0.84	0.17	0.10	0.08	0.41	− 0.02	0.84
MCP-1	− 0.11	0.31	− 0.170	0.10	− 0.10	0.34	− 0.03	0.75
PEDF	− 0.08	0.44	− 0.05	0.62	− 0.12	0.25	− 0.16	0.13
IL-6	− 0.06	0.53	− 0.03	0.77	− 0.07	0.46	− 0.06	0.52
IL-8	0.11	0.29	0.10	0.34	0.16	0.12	0.11	0.30
IL-12	− 0.03	0.74	− 0.05	0.62	0.06	0.54	0.04	0.68
IP-10	0.07	0.49	0.01	0.92	− 0.08	0.42	− 0.04	0.64
sICAM-1	− 0.27	0.03	− 0.23	0.06	− 0.29	0.01	− 0.25	0.04

*r-MBR* relative mean blur rate on the optic nerve head vessel (RRD eye/fellow eye), *sVEGFR* soluble vascular endothelial growth factor receptor, *VEGF* vascular endothelial growth factor, *PlGF* placental growth factor, *MCP-1* monocyte chemoattractant protein-1, *PEDF* pigment epithelium-derived factor, *IL* interleukin, *IP-10* interferon-inducible 10-kDa protein, *RRD* rhegmatogenous retinal detachment.

### Aqueous humor levels of sICAM-1 and optic nerve head r-MBR (RRD eye/fellow eye) in eyes with macula-on and macula-off RRD

**Figure 3:**
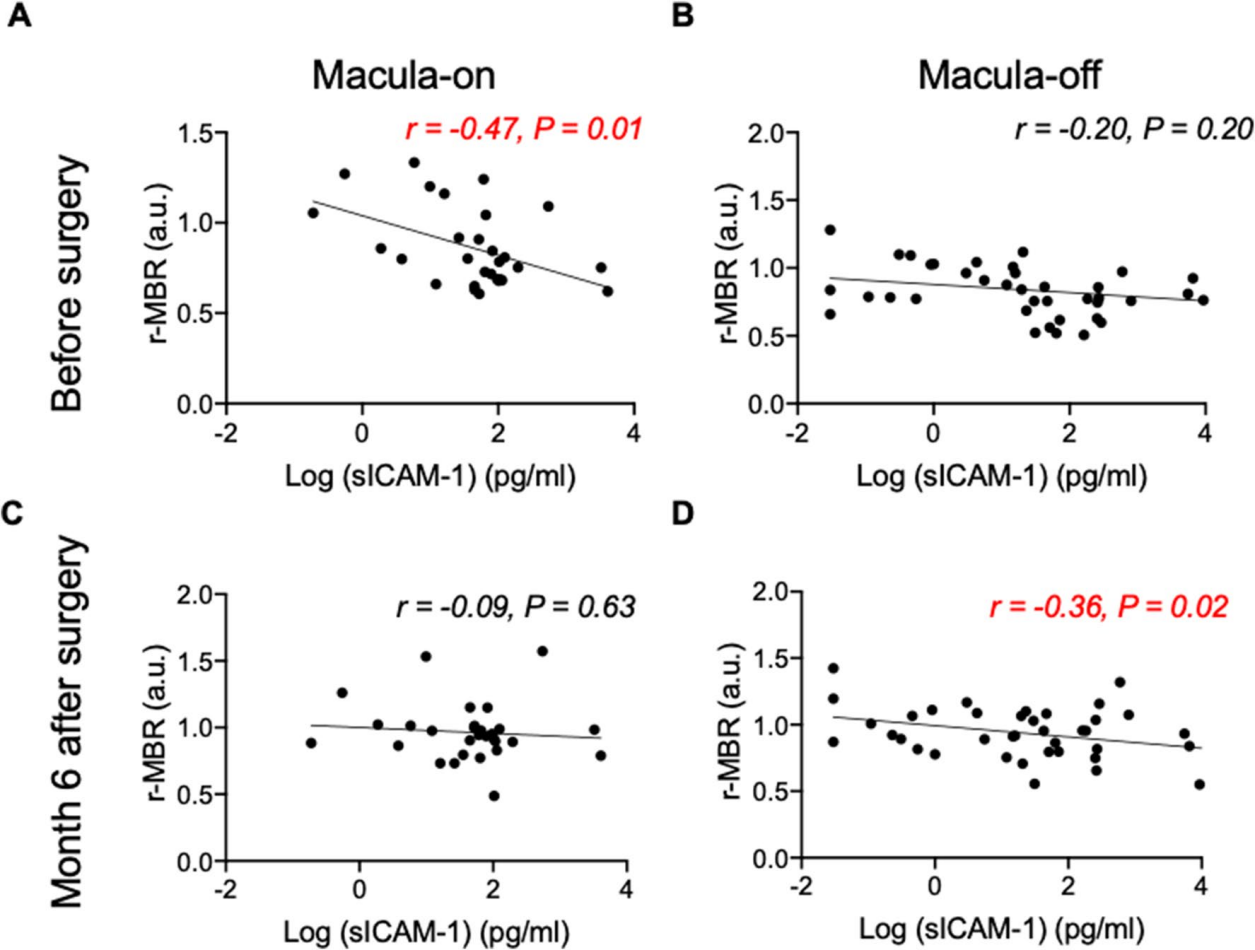
Relationship between sICAM-1 and relative MBR (r-MBR, RRD/fellow eye) in macula-on and -off RRD (**A–D**). Log-transformed levels of sICAM-1 in aqueous humor were significantly negatively correlated with vessel MBR ratio (RRD eye/fellow eye) before surgery in macula-on RRD (*r* =  − 0.47, *P* = 0.01). In macula-off RRD, log (sICAM-1) was negatively correlated with vessel MBR ratio 3 months (*r* =  − 0.36, *P* = 0.02) and 6 months (*r* =  − 0.36, *P* = 0.02) postoperatively.

To further examine the relationship between the aqueous humor levels of sICAM-1 and the time course of r-MBR in macula-on and -off RRD, we calculated the Pearson correlation coefficient before surgery and 6 months postoperatively (Fig. 3). Before surgery, we found a significant correlation in the macula-on RRD eyes (*r* =  − 0.47, *P* = 0.01), but not the macula-off RRD eyes (*r* = − 0.20, *P* = 0.20). At 6 months after 25G MIVS, log-transformed sICAM-1 levels were significantly correlated with r-MBR only in the macula-off RRD eyes (*r* =  − 0.36, *P* = 0.02).

## Discussion

In the present study, we observed the time course of retinal circulation in eyes with RRD by measuring vessel MBR in the optic nerve head using LSFG. Consistent with previous reports^4^, 25G MIVS promoted restoration of retinal circulation in macula-on RRD. Our study demonstrated for the first time that retinal circulation in macula-off RRD remained impaired after treatment with 25G MIVS and that the aqueous humor level of sICAM-1 at baseline was negatively correlated with retinal circulation in RRD eyes postoperatively.

The prognosis of visual function after surgical repair of RRD depends on the extent of macular involvement. Macula-off RRD gives rise to degeneration in neurons and blood vessels in the macula^15^. Our data also showed significant reductions in BCVA and CRT in macula-off RRD, indicating that neurodegenerative changes occurred at the macula. Retinal circulation in eyes with macula-off RRD did not recover to the levels observed in fellow eyes until at least 6 months after 25G MIVS. Accumulating evidence suggests that retinal circulation is severely impaired in retinal degenerative diseases such as retinitis pigmentosa and other inherited retinal diseases^16–18^. Macula-off RRD led to thinning of the macula and degeneration of photoreceptors. Therefore, the circumstances of macula-off RRD might somewhat replicate retinal degenerative disorders. Given that retinal circulation is highly regulated according to neuronal demand for oxygen and nutrients, we speculate that the persistence of deteriorated retinal circulation is associated with the severe neurodegenerative changes of macula-off RRD.

Recovery of the neuronal retina is also a concern after successful reattachment in macula-off RRD. In the present study, the retina remained thin in macula-off RRD compared to macula-on RRD (Fig. 1B), indicating irreversible neurodegeneration in macula-off RRD. Given that impaired retinal circulation would be expected to affect the neurosensory retina, it was surprising that there was no correlation between preoperative r-MBR and postoperative CRT. A previous clinical study using focal macular electroretinograms observed that neuronal function recovered gradually in macula-off RRD until at least 6 months postoperatively, although it did not recover fully^19^. To assess the relationship between preoperative retinal circulation and postoperative alteration of neuronal retina in macula-off RRD in more detail, a future study should employ not only optical coherence tomography measurement of retinal thickness but also focal macular electroretinograms.

Elevated levels of cytokines have been demonstrated in RRD eyes in the intraocular fluids, such as the vitreous and aqueous humor^20–22^. We found that levels of PlGF, PEDF, IL-6, IL-8, and IP-10 were more elevated in macula-off RRD than macula-on RRD. PlGF is a member of the vascular endothelial growth factor (VEGF) family and accelerates angiogenesis and inflammation^23^. To date, no study has examined intraocular levels of PlGF in RRD eyes. In contrast, intraocular levels of VEGF have been extensively examined in RRD and have consistently remained unaffected^14,22,24^. Our data further substantiate the notion that VEGF level is not altered by the RRD or its severity. PEDF exhibits anti-angiogenic effects^25^ and is essential for neuronal development in the retina^26^, and supplementation with PEDF has a significant neuroprotective effect^27^. Recently, Takahashi et al. reported that vitreous levels of IL-6, IL-8, and IP-10 were elevated in RRD eyes^24^, and IL-8 was significantly correlated with the extent of the detached area. In RRD, the role of IL-6 and IL-8 is not fully elucidated, but IP-10 was reported to be involved in photoreceptor death^22^. Although we did not examine the extent of retinal detachment in our patients, we speculate that a range of severity might account for differences we observed in the concentrations of these cytokines.

To our knowledge, the present study was the first to explore the relationship between intraocular cytokine levels and MBR in RRD. As shown in Table 2, sICAM-1 was significantly correlated with retinal circulation in RRD eyes. In central retinal vein occlusion, MBR is negatively correlated with aqueous humor levels of cytokines such as PlGF, sICAM-1, and IL-8, but not VEGF^28^, suggesting that inflammatory cytokines play a key role in the worsening of retinal circulation in eyes with central retinal vein occlusion. Our results further suggest that ICAM-1 might contribute to the deterioration of retinal circulation in cases of RRD. ICAM-1 is an adhesion molecule and promotes leukostasis in ischemic retinopathies such as retinal vein occlusion and diabetic retinopathy^28–33^, in which inflammation plays a crucial role. In the present study, sICAM-1 levels in RRD eyes were significantly correlated with IL-6 and IL-8 levels (Figure S1), indicating that sICAM-1 levels are probably related to inflammation in eyes with RRD. But neither IL-6 nor IL-8 were correlated with MBR in RRD as shown in Table 2. These results also indicate that ICAM-1, but not IL-6 or IL-8, plays a crucial role in the impaired retinal circulation in RRD. However, ex vivo or in vivo experiments are needed to investigate the direct effect of ICAM-1 on retinal arterioles and the source of ICAM-1 in RRD.

There are some limitations to the present study. First, we did not determine the extent of RRD. Whether the extent of detachment affects retinal circulation is controversial, with studies such as that by Eshita et al. indicating that extent does correlate negatively with retinal circulation in the macula^34^, and Iwase et al. reporting that MBR in the optic nerve head did not correlate with the extent of retinal detachment before surgery^4^. Second, we did not evaluate the effect of PPV itself on the measurement of MBR at the optic nerve head. Iwase et al. reported that the vessel MBR at the optic nerve head did not change between pre- and post-PPV measurements in patients with epiretinal membrane^4^. Therefore, the present results using LSFG measurement directly reflect a harmful effect of RRD on retinal circulation before and after 25G MIVS. Third, some subjects were not included in the analysis of cytokine and chemokine levels because the levels in their samples were below the measurement limits. Therefore, if a larger number of subjects are studied, we might find that cytokines other than ICAM-1 play an important role in the impaired retinal circulation in RRD. Last, our study did not measure the levels of cytokines and chemokines in the aqueous humor in eyes without RRD. Because intraocular levels of ICAM-1 are increased in other retinal disorders, such as retinal vein occlusions, future studies need to enroll a control group without ischemic retinal disorders.

In conclusion, our findings show that retinal circulation in macula-off RRD is impaired for at least 6 months after 25G MIVS. Elevations in ICAM-1 were associated with the persistence of impaired retinal circulation in macula-off RRD. Further investigation is needed to elucidate the role of ICAM-1 in the deterioration of retinal circulation in RRD.

## Materials and methods

### Ethics statement

This retrospective, observational study was approved by the Ethics Committees of Tokyo Medical University and Nihon University. All procedures were implemented in accordance with the tenets of the Declaration of Helsinki.

### Subjects

We reviewed the records of 150 patients with RRD who underwent 25G MIVS from September 2014 to June 2018 at Hachioji Medical Center, Tokyo Medical University. Patients were categorized into macula-on RRD (*n* = 63) and macula-off RRD (*n* = 87), which were defined respectively as the presence and absence of subfoveal fluid on preoperative clinical evaluation. To minimize variation in retinal circulation, participants were excluded if they had diabetes or ocular abnormalities such as glaucoma, uveitis, or a history of ocular surgery. Informed consent was obtained from all participants. We acquired the decimal best-corrected visual acuity (BCVA), intraocular pressure (IOP), and central retinal thickness (CRT) using spectral-domain optical coherence tomography (Spectralis, Heidelberg Engineering, Heidelberg, Germany). At every visit, blood pressure (BP) was monitored. For analyzing variables affecting retinal circulation, mean arterial pressure (MAP) was calculated as MAP = diastolic BP + (systolic BP − diastolic BP)/3. Ocular perfusion pressure (OPP) was calculated as OPP = 2/3 MAP − IOP. Decimal BCVA was converted to the logarithm of the minimal angle of resolution (logMAR) scale for statistical analysis.

### Surgical procedure

All procedures were performed under local anesthesia via sub-Tenon injection using 2% xylocaine. Immediately after anesthetic injection, we collected the aqueous humor with a 30G needle and stored it at − 80 °C until use. Cataract surgery was performed with phacoemulsification and aspiration (PEA) if necessary. All cases of PEA were followed by intraocular lens implantation. A 25G MIVS (Constellation, Alcon, Fort Worth, TX, USA) wide-angle viewing system (Resight, Carl Zeiss Meditec AG, Jena, Germany) was used for PPV, during which the vitreous base was shaved circumferentially as much as possible with partial scleral depression. After fluid–air exchange, conventional laser photocoagulation was performed. Filtered room air or 20% hexafluoride sulfate (SF_6_) was used as the gas tamponade.

### LSFG

Retinal circulation was measured using LSFG-NAVI (Softcare Co., Ltd., Fukutsu, Japan) as detailed previously^4,35^. In brief, the pupil was dilated with 0.5% tropicamide 30 min before LSFG, with the patient in sitting position. A fundus camera equipped with a diode laser (wavelength, 830 nm) and a charge-coupled device were used to obtain an image of a speckle pattern that was generated by scattering light from erythrocytes. The mean blur rate (MBR), which reflects the relative velocity of blood flow^35,36^, was calculated from the variation of the blurring in the speckle pattern and expressed in arbitrary units (AU). We analyzed the average MBR of the vessels on the optic nerve head using LSFG Analyzer software (version 3.2.19.0, Softcare Co., Ltd.)^4^. To normalize the MBR on the optic nerve head in RRD eyes, we also calculated relative MBR (r-MBR) by dividing the MBR value of the RRD eye by that of the fellow eye.

### Measurement of cytokines and growth factors in the vitreous

Cytokine concentrations in the aqueous humor were measured by enzyme-linked immunosorbent assay, as previously reported^37^. Briefly, 100 μL of aqueous humor per subject was applied to MILLEPLEX MAP® Human Cytokine/Chemokine Magnetic Bead Panel-Immunology Multiplex Assay (Merck, Darmstadt, Germany). The procedures were performed according to the manufacturer’s instructions. In the analyses of the relationship between cytokine levels and r-MBR, cytokine levels were log-transformed^28^.

### Statistical analysis

Data are expressed as the mean ± standard deviation. Statistical comparisons of the parameters between the groups were performed using the Mann–Whitney U-test. Two-way repeated-measures analysis of variance and post-hoc Bonferroni multiple-range test were used to identify significant differences in the parameters between macula-on and -off RRD at baseline and at postoperative visits at 1, 3, and 6 months. Pearson correlation coefficient was calculated to determine whether elevated cytokine levels affect postoperative retinal circulation. *P* < 0.05 was considered significant.

## Supplementary Information

Supplementary Information 1.

Supplementary Information 2.

## Data Availability

All data generated or analysed during this study are included in this article.
