# Supplementary material for: Role of ICAM-1 in impaired retinal circulation in rhegmatogenous retinal detachment
Source: Sci Rep. 2021 Jul 28;11:15393. doi: 10.1038/s41598-021-94993-w (PMC8319174; doi:10.1038/s41598-021-94993-w)
Supplement: Supplementary file 1 — Supplementary Information 1. [file 41598_2021_94993_MOESM1_ESM.docx]

**Supplementary Figure**. Relationship of sICAM-1 with IL-6 and with IL-8 in RRD eyes. A significant positive correlation was found between log-transformed levels of sICAM-1 and log (IL-6) (*r* = 0.36, *P* = 0.02) (**A**), and log (IL-8) (*r* = 0.26, *P* = 0.03) (**B**) in aqueous humor.
