# Supplementary figures and images for: Role of ICAM-1 in impaired retinal circulation in rhegmatogenous retinal detachment
Source: Sci Rep. 2021 Jul 28;11:15393. doi: 10.1038/s41598-021-94993-w (PMC8319174; doi:10.1038/s41598-021-94993-w)

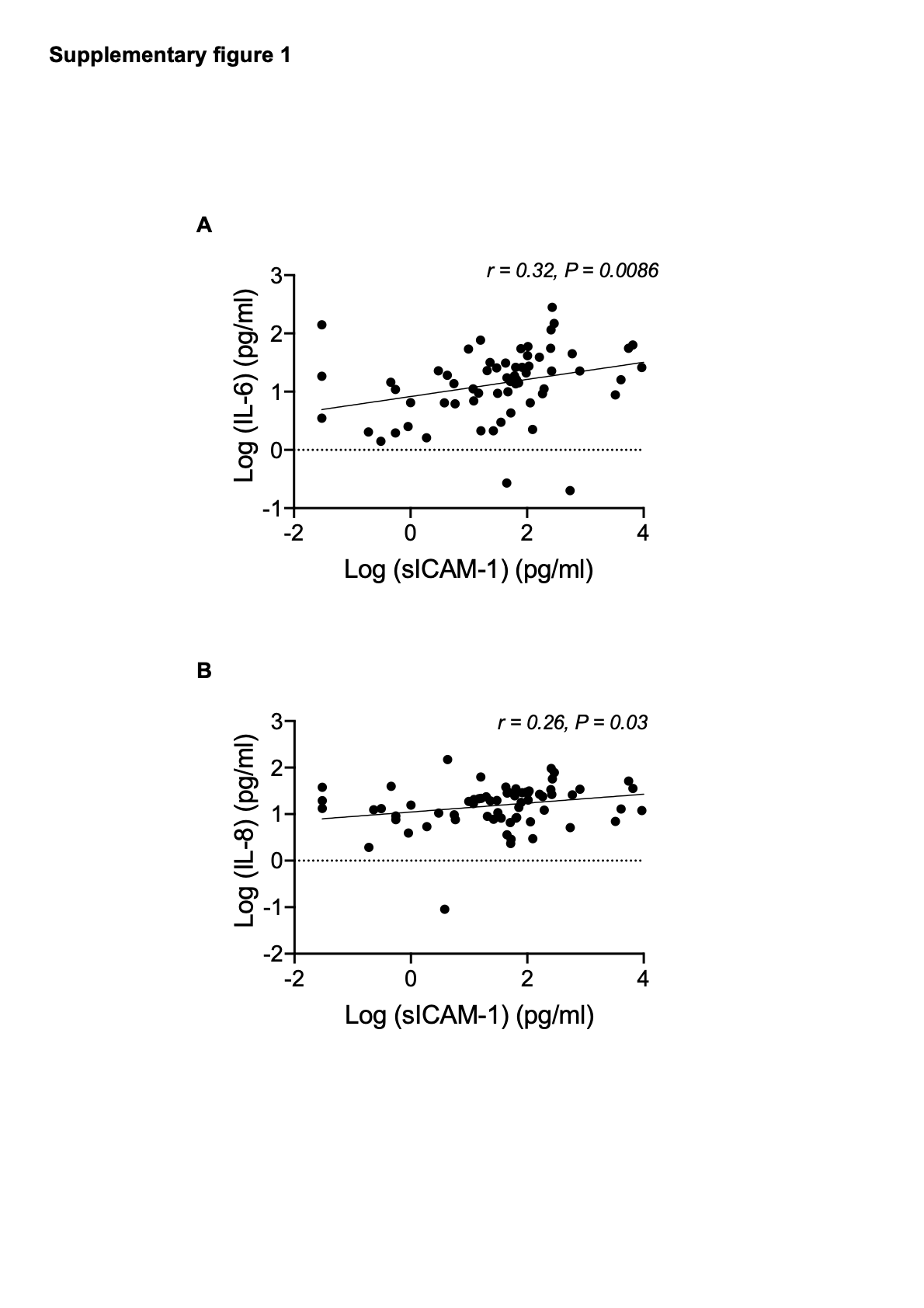

Supplement: Supplementary file 2 — Supplementary Information 2. [file 41598_2021_94993_MOESM2_ESM.tiff]
